# “I have never seen something like that”: Discrepancies between lived experiences and the global health concept of child marriage in northern Tanzania

**DOI:** 10.1371/journal.pone.0249200

**Published:** 2021-04-01

**Authors:** Susan B. Schaffnit, Mark Urassa, Joyce Wamoyi, Maria Dardoumpa, David W. Lawson

**Affiliations:** 1 Department of Anthropology, College of Letters and Science, University of California Santa Barbara, Santa Barbara, California, United States of America; 2 Department of Sexual and Reproductive Health, National Institute for Medical Research, Mwanza, Tanzania; 3 Department of Sociology II, Universidad Nacional de Educación a Distancia, Madrid, Spain; Centre for Sexual Health & HIV/AIDS Research, ZIMBABWE

## Abstract

**Background:**

The concept of ‘child marriage’ in global health distinguishes ostensibly harmful from healthy ages to marry at a universally-applied threshold of 18-years. With intensifying efforts to end child marriage, targeted communities are increasingly asked to change their perception of such marriages from relatively benign to profoundly problematic. The objective of this study is to understand how this shift in perception is navigated by adolescent girls and young women (AGYW).

**Methods:**

Using qualitative data collected in 2019 from a semi-urban community in Tanzania where marriage under 18-years is common and campaigns to end child marriage ongoing, we contrast reports of lived experiences of marriage under 18-years among AGYW to views of child marriage as an abstract concept. Thirteen in-depth interviews with AGYW, as part of a wider qualitative study, were recorded, transcribed, and analyzed using a framework analysis approach.

**Results:**

While many AGYW had heard of child marriage, the concept was routinely conflated with forced marriage, which is rare in the community, and non-marital teenage sex and pregnancy, which are common. As a likely consequence, participants disagreed on whether or not child marriage occurs locally. Furthermore, accounts of real-life marriages under 18 sometimes aligned with, but often departed from, common narratives about the purported causes and harmful consequences inherent to the global health concept of child marriage.

**Conclusions:**

We argue that engaging with diverse local views and experiences of marrying young is essential to producing culturally-sensitive, effective initiatives addressing the vulnerabilities of female adolescence.

## Introduction

Over past centuries, cultural and legal shifts in high-income countries surrounding concepts of childhood have resulted in radical changes in attitudes towards marriage under 18-years [1–3]. For example, in the United States throughout the mid-19^th^ and early 20^th^ centuries cost-free schooling proliferated followed by the implementation of compulsory schooling laws [1]. Legal ages at marriage shifted upwards during this time on a state-by-state basis, corresponding to attitudinal shifts towards children. Once seen as working members of an economic family unit, children were increasingly viewed as innocent, in need of protection, and distinct from adults [3]. Laws formalized concepts of childhood, demarcating childhood from adulthood at an 18-year threshold and attributing rights to this demographic, while simultaneously rejecting children’s ability to exercise agency by virtue of their age/development [4]. Though the adoption of this definition of childhood can be seen as the product of historical cultural and legal changes primarily in Europe and North America, this definition of legal adulthood is now widely accepted (e.g. see: [5]). With these changes, female marriage under 18-years, once considered ordinary and benign in high- and low-income nations alike, is now widely understood to be profoundly problematic and definitively harmful to girls.

As a result of changed attitudes and laws regarding childhood, governments and international non-governmental organizations (INGOs) now act to eliminate ‘child marriage’ worldwide [6]. The behavior remains particularly common in sub-Saharan Africa, where 35% of adolescent girls and young women (AGYW) marry before 18-years [7]. Actors in the movement to end child marriage typically promote a unified account of marriages under age 18-years, arguing that they have long-lasting harmful wellbeing consequences [8–11], and wider costs for economic development [12]. We refer to this viewpoint as the ‘global health concept of child marriage’. Within this framework, and in line with legal concepts of childhood [13], marriages under age 18-years are portrayed as lacking informed choice and typically being driven by parents’ and/or men’s interests at the exclusion of women’s agency, and more generally framed as a product of poverty, gender inequalities, and harmful traditions [14]. Campaign narratives routinely highlight relatively extreme scenarios, e.g. prepubescent girls forced to marry much older men by their parents [15]. Ultimately, female marriage under 18-years is seen as a lynchpin in a complex system of inequalities, which if eliminated, will lead to an improved quality of life and health for AGYW [16].

In contrast to this view of marriage under 18-years as definitively harmful, viewpoints are nuanced and varied in communities where it remains common. In many respects, this is unsurprising. The threshold of 18-years presents an arbitrary, culturally-inflexible threshold [3, 17, 18]. Yet, marriages under 18-years are not qualitatively distinct from later marriages in terms of customs and social expectations; and in many communities where early marriage is common, the vast majority of AGYW who do not marry under 18-years do so shortly after. Poverty, parental pressure, gender inequalities, and tradition certainly play into decisions about the timing of marriage all over the world [19–21], but early marriage, particularly when it occurs in later adolescence, is often viewed by community members as a solution to, rather than the root cause of, risks and limited opportunities faced by AGYW. Some girls view marriage as their primary means of gaining respect, security, or adult privileges when other paths to these ends are unavailable [19, 22–24], and AGYW are frequently active agents in the decision to marry, sometimes against the parental wishes [20, 23, 25]. Both parents and girls report that marriage can be protective against competing risks of adolescence, including early (unwanted) pregnancy, sexual abuse and infections, and continued poverty [26–29]. In other cases, marriage in adolescence is seen as neither problem nor solution, but simply normal [27]. Together these observations suggest that those most at risk of marriage under 18-years rarely consider it as the root cause of hardships they are navigating, but rather as a tool for dealing with these hardships.

The objective of this study is to understand how this disconnect and reshaping of narratives around marriage under age 18-years is being navigated by communities facing external pressure to adopt the global health concept of child marriage. This pressure is applied at multiple levels of intervention, including national-level drives for criminalization of marriage under 18-years and community-level campaigns seeking to disrupt traditions of early marriage and reframe long-held views of these marriages as acceptable or desirable for AGYW. The promotion of a unified global health concept of child marriage may effectively garner support from donor countries and change attitudes in target countries, but comes with potential negative unintended consequences as seen in other health campaigns, such as stigmatization, shame and loss of hope among target communities [30–33]. Furthermore, the inherent inflexibility of the global health concept of child marriage as a harmful cultural practice may stifle opportunity for critical reflection on the validity of concept. This leaves open the possibility that an undue fixation on abolishing marriages specifically under 18-years may ultimately limit the effectiveness of policies aiming to improve the lives of AGYW who marry both below and above this threshold.

**Fig 1:**
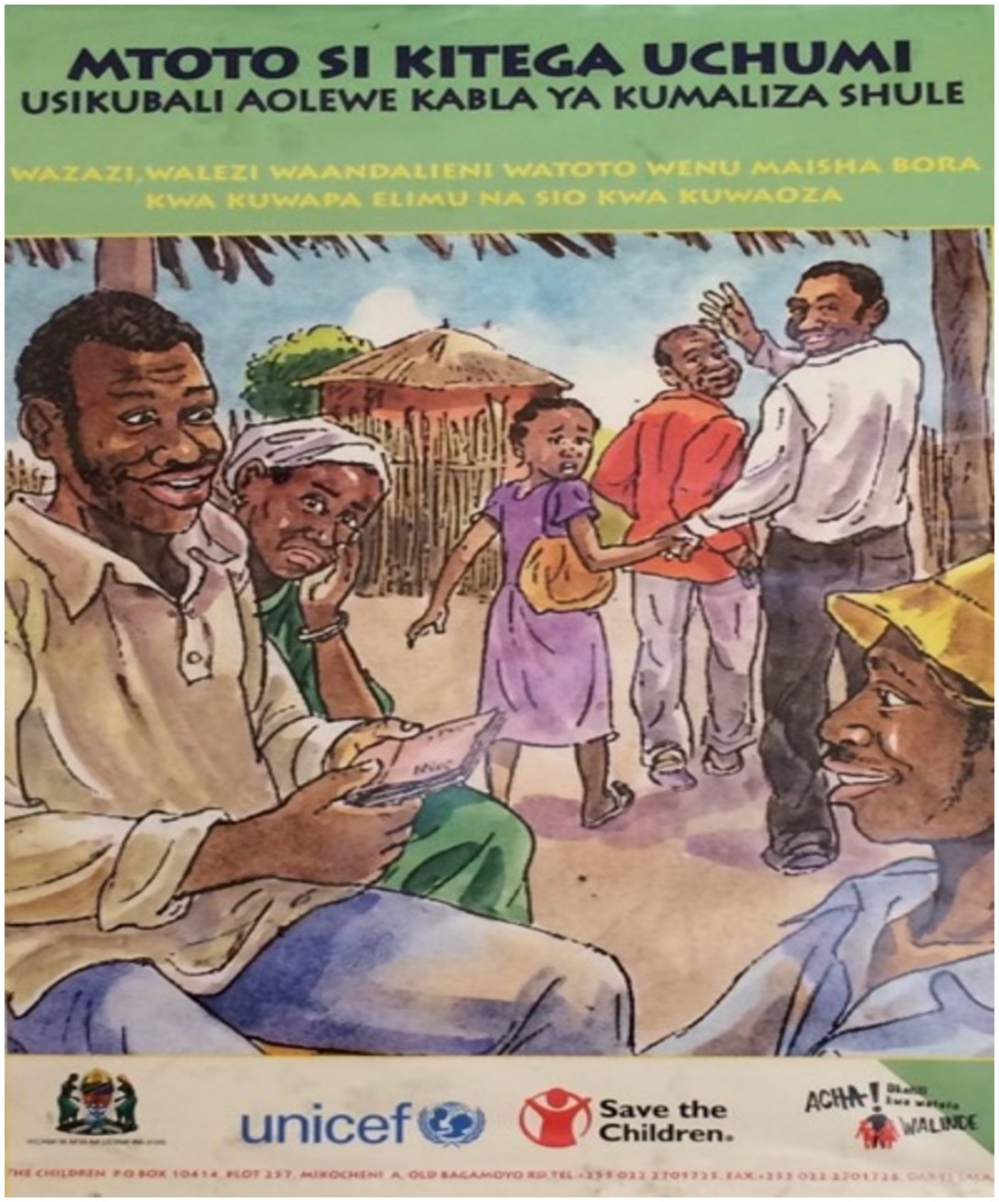
Photo of poster displayed at village government office in study site. Swahili text reads: “A child is not a commodity. Do not let her marry before she finishes school. Parents and caregivers, give your children a better life by giving them an education and do not let them marry.” Photo by: David Lawson.

To fulfil our objective, we present the results of a qualitative investigation into AGYW’s views of child marriage in a community in Tanzania currently subject to externally-driven campaigns against child marriage (Fig 1). Specifically, we contrast abstract descriptions of child marriage—its causes and consequences, and community attitudes—to real-life accounts of marriages under 18-years. We also identify the content and sources of information on child marriage that AGYW have received.

## Methods

### Study context

This study took place within the semi-urban town center of Kisesa Ward, in Mwanza Region, home to an ongoing Health and Demographic Surveillance Site (HDSS) managed by the Tanzanian National Institute for Medical Research (NIMR; [33]). The area is rapidly urbanizing and home to over 42,000 primarily Sukuma residents (the largest ethnic group in Tanzania). Traditionally agropastoralists, Sukuma people now occupy diverse professions including skilled and unskilled labor. This is particularly true in the semi-urban study site where small plot sizes do not allow for extensive cultivating or grazing, as would be possible in the less densely populated areas surrounding the town.

Marriage encompasses both formal and informal unions. Formal marriages usually include a transfer of bridewealth from a husband to his parents-in-law, while informal marriages only require cohabitation. While informal arrangements are accepted as ‘real’ marriages, the bounds between unmarried relationships and marriages are often blurred. Marriages of minors are declining [34] but remain common; in 2017, 24% of young married women did so before age 18-years and less than 2% before age 15 [30] (compared to 31% married before age 18 years and 5% before age 15 years in the country as a whole [7]). Marriages under 18-years are more common in surrounding villages where populations are less dense and agropastoralism more common. Marriages are generally initiated by the marrying couple, as opposed to arranged by parents or other relatives, even when the marriages occur at young ages [23]. Young people are motivated to marry for the social benefits of marriage [23], but also due to constraints imposed by wider risks of adolescence and structural factors like poverty. Both divorce and re-marriage are common in this area [35], as are pre- or extra-marital pregnancies and births [23] and transactional sex [36].

At the national level, recent external pressure to increase legal ages of marriage in Tanzania means that child marriage is a salient topic, frequently discussed in local media. In 2016, the legal age of marriage was raised from 15 to 18-years for girls. At the same time, a law banning sex or marriage with school girls came into effect [37]. In 2017, a legal challenge was raised against the newly proposed minimum age of marriage due to incompatibility with customary laws [38]. The law targeting sex and marriage to schoolgirls remained unchallenged. In October 2019 (2 months following data collection) the change to the legal age minimum for marriage officially came into effect when the appeal against the 2016 change was dismissed [39]. At the local level, the study community has been exposed to multiple efforts to shift local attitudes on early marriage (e.g. Fig 1).

### Data collection, processing and analysis

In June-August 2019, we carried out 15 Focus Group Discussions (FGDs) and 33 in-depth interviews (IDIs) with AGYW, parents of AGYW, young men, and community leaders. For the purposes of this study, we use data from the 13 IDIs conducted with AGYW specifically. AGYW are often the targets of ‘empowerment’ interventions that aim to alter attitudes towards child marriage and provide tools to ‘fight’ marriage in adolescence [40, 41]. As such, we expect that AGYW will have been exposed to and aware of the global health concept of child marriage via mass media and local actors in the movement to end child marriage. Additionally, given the commonality of young marriages [30, 34], we also anticipate that AGYW will have firsthand knowledge of their own and/or peers’ experiences of early marriage. Despite the modest sample of in-depth interviews (IDIs) with AGYW, we emphasize that our wider characterization of the study community does not rest on the IDIs in isolation. Rather, we build upon our previous work on early marriage and adolescent behavior in this same population [23, 29–32].

IDIs focused specifically on ‘child marriage’. They were designed to establish participants’ (a) knowledge of child marriage as a concept, (b) their sources of knowledge, (c) experiences of, and (d) views/attitudes on child marriage and marriage under 18-years. In contrast, FGDs focused on risks and opportunities of adolescence more broadly [29]. IDI participants were recruited by facilitators from the FGDs conducted with AGYW, aged 15-18-years and 19-24-years, attempting to balance the number of married and unmarried participants across the two age groups, and targeting AGYW known to have married before age 18-years. FGD participants were selected using snowball methods, starting with a ‘seed’ participant who matched the desired characteristics for the FGD [29].

Interviews were conducted by female social scientists trained by the authors using an IDI guide (see S1 File). Interviews were conducted in Swahili or Sukuma depending on the desires of the participant and audio recorded. Most interviews lasted about 45 minutes always in a private area away from potential listeners. All participants were compensated for their travel with 5,000 Tanzanian Shillings (~2.50 US dollars) in accordance with NIMR guidelines.

Recorded IDIs were transcribed verbatim by facilitators and translated by NIMR employees. The data were analyzed using a framework analysis approach, identifying themes arising from the research questions and allowing new themes to arise from participants’ narratives [42, 43]. Data were coded in NVivo 12 by two researchers and then studied, organized and interpreted with regards to the study’s aims. Pre-arranged themes were discussed and clarified by both coders prior to coding the data. Themes arising were discussed by the coders throughout the process. Differences were resolved through conversation. In organising the data, special attention was paid to distinguishing discussions of ‘child marriage’ in the abstract from stories of lived (or observed) experiences of the marriage of minors. Statements not linked clearly to an individual were considered abstract, while stories pertaining to specified events/people were considered lived experiences. Within conceptual discussions of ‘child marriage’, data were organized by several questions: *“What is child marriage*? *What do you think about child marriage*? *Why does it happen*? *What are the consequences*? *Where/what did you hear about child marriage*? *Does it happen here in this community*?*”* Similar questions about why marriages happen and with what outcomes were considered for lived experiences.

### Ethical considerations

Written parental/guardian consent and participant assent was obtained for minors at recruitment, and participant consent was obtained from adult participants immediately prior to interview. Participants were read a consent statement which outlined the study’s goals and risks, and the benefits of participation. Following this, participants were allowed time to ask questions (or decline participation) and were given a hard copy of this statement along with contact information for NIMR should they have questions or concerns arising at a later time. Identifying information has been removed from all data presented below including community members’ names, town and neighbourhood locations, names of NGOs, and any other details that could identify a study participant or fellow community member.

This study was granted ethical approval by the Tanzanian National Institute for Medical Research Lake Zone Institutional Review Board (MR/53/100/595), Tanzanian National Ethical Review Committee (NIMR/HQ/R.8a/Vol.IX/3104), and the University of California Santa Barbara Human Subjects Committee (2-18-0993).

## Results

Participants ranged from 17 through 24 years old and included six married, one divorced, and six unmarried AGYW (Table 1). Five of the seven ever-married AGYW married before age 18-years.

**Table 1:** In-depth interview participant characteristics.

Age	Marital Status*	# of children	Married <18-years?	Heard of ‘child marriage’?	ID**
17	Divorced	0	Yes	Yes [TV]	17yo_m_0
18	Unmarried	1	-	Yes [TV and radio]	18yo_u_1
18	Unmarried	0	-	Yes [local NGO]	18yo_u_0a
18	Unmarried	0	-	Yes [local seminar]	18yo_u_0b
18	Married	2	Yes	No	18yo_m_2
18	Married	1	Yes	No	18yo_m_1
19	Unmarried	0	-	Yes [school]	19yo_u_0
22	Unmarried	1	-	No	22yo_u_1
23	Unmarried	0	-	Yes [school]	23yo_u_0
23	Divorced, Remarried	2	Yes	Yes [news]	23yo_m_2
23	Married	1	No	Yes [local NGO]	23yo_m_1
24	Married	3	Yes	No	24yo_m_3a
24	Married	3	No	Yes [informal]	24yo_m_3b

*all married participants were in monogamous marriages

**ID is used to identify speakers of quotes presented below

### The concept of child marriage was widely known, but ideas about what it represents were muddled

Most participants had heard of child marriage, through formal channels like television and radio, local informational campaigns and schooling, or indirectly from community members:

“We had a girls’ group [at an NGO]. We used to discuss about childhood marriages, and we used to be told to have self-awareness and confidence.”

[18yo_u_0a]

“There is no specific person. People just talk about it [child marriages] when they meet… They will talk about somebody`s daughter who was married but now is divorced.”

[18yo_u_1]

Although participants had usually heard of child marriage, pressing participants to define it revealed that it is often conflated with early/transactional sex, teenage pregnancies or forced marriages, rather than simply marriage under 18-years:

“*Interviewer*: When you hear someone saying that [child marriages] what do you understand?

*Participant*: I understand that they are doing sex while they still young.

*Interviewer*: What do you think the word marriage means?

*Participant*: I know that when [someone gets] married, they have a ceremony. That is a marriage.

*Interviewer*: So, what is a child marriage?

*Participant*: I don’t understand those. I just understand that, maybe they are just doing sex at childish age.”

[18yo_m_2]

Child marriage was discussed by many participants as specifically forced or arranged marriage:

“It [child marriage] is a sexual abuse when you force a child who is under 18-years old to get married without her consent, so therefore it is like you have sexually abused her”

[18yo_u_1]

Perhaps as a consequence of this confusion, there was disagreement about whether child marriage unequivocally happened in the study area. Some participants felt that it was a behavior that took place elsewhere in Tanzania, but not locally.

“I normally hear this [child marriage] happens in the village, but here? No, I never heard of it.”

[23yo_m_1]

“I have never seen something like that”

[23yo_m_2]

Other participants thought that child marriages might happen in their community but were limited in number or type. For example, participants noted that it was not marriage in adolescence that was common, but pregnancy or transactional sex or sex work:

“I haven’t heard anything at the moment but for girls in [this town], most of them do not rush to get married…. They do not rush to get married but rather sell their bodies for money. I saw a fellow student selling herself at the bus stand.”

[17yo_m_0]

“For [this community], here there are none. They might not be getting married at younger ages but they can be impregnated and bear their children while they are at home, so that exists.”

[23yo_m_1]

### Contrasting with abstract stories, real-life accounts of child marriage focused on agency not coercion

A common view was that child marriages were the result of parental pressure, with the term “forced” used frequently. Participants explained that parents may force their daughter to marry after she ends her education, through completion or drop out, and in many cases, parents were portrayed as greedy, forcing their daughters to marry in order to receive bridewealth:

“That can happen. Maybe you failed in school or you were not lucky to go to school and then your parents arrange for a person to marry you and so you will get married without your consent.”

[18yo_u_0b]

“Greed for wealth. Greed is what makes them [parents] force their children to get married early. They think that the bridewealth that they get will help to solve their problems at home, but they are only bringing big problems to their child.”

[23yo_u_0]

Intuitions about parental force driving child marriage aligned with information about child marriage being disseminated in their town. A participant recounted how at a local NGO she heard:

“To be married at younger age might be because of parents’ influence. They need money, cows, like in the village they want cows, so they think [it is best] to wed their daughter at younger age so as to get the bride price and meet their needs at home.”

[23yo_m_1]

In stark contrast to abstract discussions of child marriage, accounts of real-life marriages under 18-years generally emphasized that marriages were the result of girl’s own agency. For example, this married 18-year-old woman decided to marry at age 15-years:

*“Interviewer*: Please tell me the process for you to get married. How was it?

*Participant*: (Laughing) I just wanted to get married. I am matured.

*Interviewer*: Who did you tell that you are matured and you want to get married?

*Participant*: Who? I just decided by myself…He [her husband] saw me at the weekly market … and he said that he is looking for a woman to marry. He came to my home, and I talked to him first. Then we agreed with each other [to marry]…He told me ‘you just come [to his home]’.

*Interviewer*: I want to know the process of getting married.

*Participant*: Just like that. He brought bride price. They [my parents] accepted everything. We continued to stay together until today.”

[18yo_m_1]

Another married 18-year-old woman, noted a neighbor who decided to discontinue her education and get married:

“She was in class seven and passed [~14-years-old]. After she passed, she said ‘I am not studying [anymore]. Let me just stay at home, I have the education. Let me stay at home and settle.’…So she stayed at home and she got married and she is just living good life.”

[18yo_m_2]

Female agency in the decision to marry was sometimes recounted in terms of a girl’s love and desire:

“No one convinced them [to marry]. They decided that themselves…. [A] friend of mine was tempted to be with her lover and they got married without even telling her parents. She eloped with her lover. They decided to get married because of their own desire.”

[18yo_u_1]

Only one participant spoke of a real-life experience relating to forced marriage. Speaking about her own experience, she told of how her father and grandfather attempted to force her to marriage when she was about 16-years-old. The man she was meant to marry brought a bridewealth payment to her relatives, but she told him they could not get married:

“I talked to him [the prospective husband] and I told him that I have no plan to get married. If he wants to marry, he should find another woman to marry and that’s what happened. The bride price was returned.”

[22yo_u_1]

Beyond parental coercion, other reasons were presented for why child marriage occurs. In each of these cases, discussions of child marriage in abstract terms were largely mirrored by descriptions of lived experiences. Chief among these proposed drivers was pregnancy:

“*Interviewer*: So, can a pregnancy cause a girl to get married?

*Participant*: Yes…If the one who impregnated her has the direction then she will be married. He will stay with her; he will take you to his home and stay with you”

[18yo_m_1]

“I didn’t decide [to marry at 14-years-old] but I was pregnant… It is better I tolerate this man and settle.”

[18yo_m_2]

In addition, constraining situations and lack of alternatives were identified as important drivers of early marriage. One 18-year-old woman told the story of her friend who married at 17-years-old. Her friend said she married due to problems at her family home:

“[She decided to elope because of] how she used to live at her home. She used to tell me that she was being mistreated. It was noise at home all the time. Her parents and brothers used to insult her. She insisted that she was fed up, saying that it was chaos at her home every day.”

[18yo_u_0a]

In some cases, marriage was positioned as a tool to overcome a lack of resources or unrest at girl’s home with men taking advantage of AGYW’s needs:

“…girls need pads and you find one has no ability to even get a pad. The needs are a lot like lotion and many others. So, you find when a girl lacks those basic needs. She starts looking to the other side. So, you find she involves herself with underage marriages.”

[24yo_m_3b]

“For instance, if you are a student, he [a man] will use the problems that you have to convince you [to marry him] and then you will feel like you had been wasting time all along [with school] and then you will get married.”

[18yo_u_0b]

### Child marriage was perceived to have harmful consequences

Even with muddling marriage, pregnancy, and sex, most participants expressed negative attitudes towards child marriage as a concept, frequently linking disapproval to perceived undesirable consequences. First, by far the most commonly cited negative outcome, was the instability of child marriages:

“She will still think as a child and not as an adult, so it is likely she won`t last long in her marriage because she doesn’t even know what a marriage is.”

[18yo_u_1]

“The consequences [of child marriage] do exist because you may find yourself entering early marriages then at the end of the day the marriage fails… You just remain in streets and wander with no direction you find yourself being a street child…. Maybe he [her ex-husband] has already married another woman and he can’t care for you anymore so you find your life becoming miserable.”

[22yo_u_1]

This perception was reflected in participants’ accounts of early marriage in their community that ended in divorce. Without quantitative data, it is not clear if these stories are reflective of high divorce rates in the area generally [35] or is specific to marriages entered at young ages.

Second, participants also identified costs associated with sexual and reproductive health, including negative consequences of children born from early marriages, and the risk of sexually transmitted infections:

“There are so many problems that come with child marriages, for example when the child is married under the age of 18, when they get pregnant and are about to give birth, the uterus, or eggs, or pelvis I don’t know, is usually not fully expanded so it becomes a problem since many children die during childbirth.”

[23yo_u_0]

“Don’t expect him to be faithful to you because your mind is still immature so you can’t give him enough good advice. So, when he starts seeing other women its likely he may get sexually transmitted diseases and in turn transmit them to you.”

[18yo_u_1]

Though commonly mentioned as a consequence of child marriage, adverse sexual and reproductive health outcomes rarely featured in participants’ telling of lived experiences. In contrast, pregnancy was often cited as a cause, not consequence of marriage under 18-years (see above).

Third, negative sentiments surrounding child marriage often centered around abuse that was seen to follow marriages of young people. Participants saw minors as poorly equipped to deal with abuse:

“What I know is when someone is married young, they will face gender violence. They will be mistreated by in-laws or by the husband himself. Those who are married in villages go on to herd cattle, they are not even allowed to meet with fellow women to expand their minds, they don’t have any businesses, so they go through that kind of violence.”

[23yo_m_2]

A different participant noted that the husband would abuse his wife specifically because she was so easily convinced to marry him at a young age:

“[If] a man marries you while you are still young and convinces you to run away with him without your parents’ approval, he won’t respect you as his wife. He will despise you and think if he was able to convince you to run away with him, maybe one day someone else will be able to convince you as he did to run away from him. So, he will start being angry at you, beats you up. The torture begins.”

[18yo_u_1]

Marital disharmony and abuse also came up in lived experiences. A woman who married at age 17-years spoke both of the abuse she experienced with her first husband and the healthier relationship she entered at a later date:

“The marriage was too much for me; the man I married was a dictator and he verbally insulted me and mistreated me. I had enough so I went to my sister who welcomed me and asked why I could not stay with my husband; I told her married life was hard…I stayed with my sister while raising my firstborn. In the end I felt mature enough at twenty and got married again. This marriage is different because when I entered it, I was mature enough. My life with my current husband is good.”

[23yo_m_2]

Finally, participants associated negative viewpoints of child marriage with reputational costs. Notably, reputational costs were often linked to divorces rather than marriage itself:

“Because they might say that she was divorced from her husband maybe because she had too many men or she didn’t have good manners, even when she returns to her family, they will start talking negatively about her.”

[19yo_u_0]

In some cases, real stories of marriages under 18-years also suggested that some AGYW face reputational costs due to their marriages, at least among their friends and neighbors retelling the stories. One participant spoke of her cousin who had married before age 18-years-old. Notably, this marriage caused reputational costs because the girl married ‘free of charge’ (i.e. eloped without a bridewealth transfer), not specifically because she married young:

“She is so embarrassed to come home [following her elopement]… There was this day that a certain girl was plaiting my hair and asked if [my cousin] was married… she said that she heard people saying that she had gotten married. I told her that I do not like rumors…So they [people in the community] confirmed that she has truly got married and were saying that she has really brought shame to our home because she got married free of charge and so she has embarrassed our family… It was a big shame in our streets and village.”

[18yo_u_0a]

Another woman who married at 17-years-old discussed feeling shame following her marriage, though in this case she specified that the shame was associated with having to leave school due to pregnancy rather than her marriage per se:

“I felt really bad [that I got pregnant and discontinued school] because I also humiliated my family. So I did not feel great at all.”

[24yo_m_3a]

### Positivity about early marriages was also notable

Though participants expressed various negative sentiments surrounding child marriage there were also cases of balance or nuance. One participant distinguished the harmful consequences of ‘bad’ elopements from the more acceptable outcomes of ‘proper’ early marriages:

“You know there is a difference between eloping with a girl and taking her from her family in a proper way where the parents and family will be happy. Even if you have problems in future, they will be solved [if you married the proper way]. But getting married in secrecy [i.e. eloping] … is what brings about problems. Because you may fight with your husband and lack somewhere to run to for help especially if you ran away from home; some parents are so hard at forgiving…”

[18yo_u_0a]

In other cases, participants identified positive outcomes of marrying early, even when they held generally negative views towards the behavior:

“The nice thing about it is having a family, getting married with a family full of love and God fearing, that is the fun of getting married early and that is why some want to get married early.”

[23yo_u_0]

Another woman identified a more tangible economic benefit of marriage in adolescence:

“You might get an income or the man [her husband] may open up a business for her, and she might make her own income from that business and not be dependent on the man, she might even be able to help her parents and the community at large.”

[23yo_u_0]

While these expressions of positivity and nuance were relatively uncommon when discussing child marriage in abstract terms, they were much more commonly reflected in how stories of lived experiences were told. For example, a married 18-year-old participant told of her increase in economic power following her marriage at 15-years-old:

“*Participant*: I have started to cultivate land and to buy things [after I got married].

*Interviewer*: Which things have you bought?

*Participant*: Cows.

*Interviewer*: Whose cows?

*Participant*: For the two of us—my husband and I.

*Interviewer*: Another thing [that has changed since you married]?

*Participant*: We are cultivating land; we are harvesting and selling [produce]. Before I got married, I was young. Where could I get the money?

*Interviewer*: Other changes?

*Participant*: Nothing, I think it is just normal.”

[18yo_m_1]

Another woman noted that her early marriage brought her companionship:

“You just stay with your husband, and you advise each other. You see, this is my advantage.”

[18yo_m_2]

## Discussion

We set out to explore how a semi-urban community in Tanzania is navigating the reframing of marriage under 18-years from benign to harmful. Although most participants had heard of child marriage there was confusion surrounded the term’s meaning. Confusion could stem from the generally loose concept of marriage itself. The term marriage encompasses a variety of behaviors in this community, including unions marked with a traditional ceremony (usually an exchange of bridewealth), a church or other religious ceremony, a legal ceremony with documentation, cohabitation, or a combination of these things. As such defining when a person is married is a more fluid concept [44] than often implied by global health narratives, which tend to conceptualize a readily assignable age and date to union formation. Further, unlike a comparison between monogamous and polygynous marriage, marriage before age 18-years is not qualitatively distinct from marriages at older ages in terms of associated customs and expectations. As such, the classification of child marriage as a distinct practice—inherent to the global health categorization of AGYW as ‘child brides’ and child marriage as a ‘harmful cultural practice’—may be counterintuitive in communities where marriages under 18-years cannot be distinguished from remaining marriages that often happen just above this threshold.

Reflecting this loose concept of marriage, participants’ discussions of child marriage overlapped frequently with teenage pregnancies, and (transactional) sex. Rather than exemplifying lack of understanding, this conflation highlights the interconnectedness and flexible causal links running between these behaviors. Sex can lead to pregnancy, which can incentivize marriage. Marriage is a valuable institution [23, 45] and traditionally includes a bridewealth payment transferred from a husband to his parents-in-law. Bridewealth payments are meant to ensure the delivery of children [32] and serve as a sign of respect to the bride and her family [30]. Increasing, though still early, ages of marriage in this community [34] independently of ages of first sex, mean that sex and marriage have become increasingly disassociated, leading to a situation where transactional sex may be seen as a variation of bridewealth, exchanging goods for sex, though still distinct from marriage [45, 46]. Complicated and contradictory values surrounding transactional and non-marital sex [45], mean that sex is sometimes conflated or even synonymous with marriage, as seen here, but other times marriage is conceptualized as a tool for mitigating risks of unwed sex in adolescence. As such, the interconnected nature of sex, pregnancy, and marriage are reinforced by both biological mechanisms and social context, leading to the confusion we find around defining ‘child marriage’.

To some extent, participants’ abstract discussion of child marriage corresponded to both the global health concept of child marriage and local AGYW’s accounts of lived experiences. For example, participants commonly cited divorce as an undesirable consequence of child marriage. Divorce was also frequently mentioned in stories of lived experiences of marriage under 18-years and are known to be common in the study area of [35]. Pregnancies also featured as a driver of marriage in both accounts of real adolescent marriages and abstract discussions of child marriage.

In other ways, lived experiences of marriage under age 18-years existed outside of the confines of common narratives inherent to global health portrayals of child marriage. Most notably, parental coercion was presented as a cause of child marriage and some participants cited that they learned this through media and contact with NGOs. The forced marriage narrative is indeed central to the global health concept of child health. The terms child and forced marriage are commonly used interchangeably reflecting the legal view that minors inherently cannot give informed consent [13], and imagery commonly depicts marriages of young people as forced (Fig 1; see also [15]). Yet, forced marriages rarely featured in lived experiences of adolescent marriage, with the exception of one attempted forced marriage which notably the participant refused in a demonstration of her agency. This is corroborated by previous studies of early marriage in Tanzania, including in this town, which highlights agency in the decision to enter marriages ([22, 29, 30, 47]; and also in other areas of sub-Saharan Africa [48]) and to participate in sexual activity [49]. For example, regardless of age of marriage, the vast majority of married women sampled in this town previously reported choosing their own partner [30]. The methods of the current study cannot tell us whether participants’ view that child marriages were caused by force originates specifically from narratives and imagery presented by mass media, NGOs, and/or informational campaigns addressing child marriage, i.e. the global health concept. Nevertheless, the notable rarity of forced or even arranged marriages in this community contrasted with the commonly repeated narrative of forced marriage is certainly suggestive of such a link.

Our findings complement wider investigations into early marriage in this community that cast doubt on the universal applicability of the global health concept of child marriage [23, 29, 30]. Here, we consider the implications for global health campaigns of these areas of divergence. On a practical level, campaigns to abolish child marriage will not have the desired impact of increasing ages at marriages if community members do not identify their own behavior, or the behavior of their peers, as relating to the concept of child marriage they hear about on TV/radio, in classrooms or through direct engagement with interventionists. This situation appears to characterize this study population. Marriage under age 18-years is common [30, 34] and yet many did not believe that child marriages occurred in their community. Instead it was viewed primarily as something that happened in other communities and regions. These contradictory beliefs could stem from confusion about what child marriage is, or denial inspired by a desire to distance themselves from the behavior due to social desirability bias. Alternatively, confusion could reflect disconnects between global health concepts of child marriage and lived experiences of marriages at young ages, especially with regards to the common assumption that child marriages are inherently forced marriages.

The use of popular global health narratives surrounding child marriage also raises ethical considerations, including questions of culpability. Global health narratives frequently promote the idea that parents cause child marriage for their own personal financial gain via coercion of daughters. Placing the blame for the ‘bad’ behavior on individuals underplays the role of local histories and structures which can incentivize ostensibly unhealthy behaviors, like marriage in adolescence [50–53]. This can be seen to play out in observed contrasts between global health concepts and lived experiences of early marriage recorded in this study and elsewhere. For example, in direct contrast to a local NGO’s narrative that parents promoted early marriages for selfish aims, Maasai parents in Kenya who were unable to provide daughters an education due to socioeconomic constrains felt that marriage provided the greatest potential for a bright economic future [26]. Thus, parents were the victims of inequitable structures constraining their daughters’ opportunities [26]. The common emphasis on parents’ roles in causing child marriage “deflects attention from human rights abuses that are not perpetrated by individuals but, rather, by economic, political, or social forces at large” ([26] p 638), forces which are often perpetuated by the actions of countries creating and funding global health agendas.

### Strengths and limitations

Our study makes use of rich qualitative data and sits upon a strong foundation of previous work guiding our understanding of the local context. Even so, there are also several limitations. First, we focus on the voices of AGYW but the majority of the IDI participants were young women as opposed to adolescents, limiting our ability to detect different viewpoints between younger adolescent girls and young women. That said, several of the IDI participants were married as adolescents and thus we expect that the data are meaningful for addressing our study objective. Second, we rely on a small number of IDIs which may mean that we were not able to reach saturation. However, confidence in our findings is reinforced by our previous work on female adolescence and marriage in this population [23, 29, 30, 45, 54]. Third, recruiting IDI participants from FGDs could have biased in the IDI discussions if FGDs somehow primed participants for the topics to be covered. However, FGD topics were sufficiently different from IDI topics (the topic of marriage was present in FGDs, but ‘child marriage’ was not discusses as such) that we do not have a strong reason to suspect this to be the case. Finally, given that ‘child marriage’ could be a sensitive topic our data may have been subject to social desirability bias. That said, FGD and IDI facilitators were trained to not display moral judgments about any views or behaviors reported surrounding marriage. Further, the departure of individual stories of early marriages from the global health concept suggests that participants were comfortable sharing views and experiences that departed from any perceived ‘correct’ answer.

## Conclusions

We document confusion about the meaning of ‘child marriage’ in this community. We also find that negative attitudes towards child marriage as a concept were contrasted with more nuanced and variable experiences of AGYW. While the source of these discrepancies can only be speculated, engaging with this diversity of experiences will be key to creating more ethical and effective interventions promoting healthy transitions to marriage. This engagement should include applying greater nuance to depictions of child marriage in campaign materials and interventions to ensure the global health actors and target populations are on the same page. Doing so may entail acknowledgment that sex and childbearing do not always take place within marriages, marriage in itself is transient concept, and that female agency and autonomy can exist outside of the legal boundaries of adulthood. We advocate for further research critically examining the assumptions of the global health concept of child marriage. At the very least, researchers and policy-makers alike should remain open to the possibility that discrepancies between the narratives of global health and target populations surrounding behaviors like child marriage may be due to differing rationalities and lived experiences, rather than naive misunderstandings or ignorance of communities deemed at risk.

## Supporting information

S1 File Interview guide. (DOCX)
