# Supplementary material for: “I have never seen something like that”: Discrepancies between lived experiences and the global health concept of child marriage in northern Tanzania
Source: PLoS One. 2021 Apr 1;16(4):e0249200. doi: 10.1371/journal.pone.0249200 (PMC8016342; doi:10.1371/journal.pone.0249200)
Supplement: S1 File — (DOCX) [file pone.0249200.s001.docx]

**Mwongozo wa Mahojiano ya kina**

1. Overview

**Mtazamo**

This is a guide for In-depth Interviews (IDI) which will take place in Kisesa. The aim of the IDIs is to capture different individuals’ knowledge and experience of, and opinions on what is sometimes called ‘child marriage’. Each IDI will last up to 1.5 hours.

**Huu ni mwongozo wa mahojiano ya kina ambao utatumika katika Kisesa. Kusudio la kufanya mahojiano ya kina ni kupata elimu na uzoefu wa kila mtu, na maoni katika kitu kinachoitwa ‘Ndoa za Utotoni”. Kila mahojiano ya kina yatachukua muda wa saa 1.5.**

1. Main Objectives

**Malengo makuu**

Each IDI seeks to understand the viewpoints of community members’ views on early marriage in their village in Kisesa, Tanzania. We wish to know:

**Kila mahojiano ya kina yanatafuta kufahamu maoni ya wananchi katika jamii’ maoni juu ya ndoa za mapema katika vijiji vyao, Kisesa, Tanzania. Tunataka kufahamu:**

- What do participants know about what is sometimes called ‘child marriage’?
- **Je, ni nini washiriki wanachofahamu kuhusu kitu kinachoitwa ‘ndoa za utotoni?**
- How did they learn about the topic?
- **Je, walijifunzaje kuhusu mada hiyo?**
- Have they experienced or do they know someone who married before age 18 years?
- **Je, wamepata uzoefu au kuna mtu wanayemfahamu alioa/olewa kabla ya kufikisha umri wa miaka 18.**
- What are their opinion on early marriages?
- **Je, wana maoni gani kuhusu ndoa za mapema?**

1. Instructions for Facilitator

**Maelekezo kwa Mwezeshaji**

At the start, please discuss the study’s aims and objectives, and the IDI’s importance in contributing to the overall project. Remind the participants that the interview is completely confidential in nature and their names will not be used in any reporting.

**Anza, tafadhari kwa kujadili makusudi na malengo ya utafiti, na umuhimu wa mahojiano ya kina yanavyosaidia/changia kwa ujumla katika mradi. Wakumbushe washiriki kuwa mahojiano kwa asili yake huwa ni siri na majina yao hayatatumika kwenye taarifa yoyote itakayoandikwa.**

Make sure the interviewee knows that their opinion and experiences are extremely important to the project. The questions below are meant to guide the interview, not to limit it, and there are no right or wrong answers. The interviewer should ensure key questions are explored during the discussion. However, the interviewer may elaborate on questions or change the sequence, based on the interviewee’s responses.

**Hakikisha mhojiwa anafahamu kuwa maoni na uzoefu wake ni muhimu katika mradi. Maswali hapo chini yana maana ya kumwongoza mhojaji, na sio kumpa mipaka, na hakuna majibu sahihi au majibu mabaya. Mhojaji anatakiwa kuhakikisha maswali yote muhimu yanafafanuliwa wakati wa majadiliano. Hata hivyo, mhojaji anaweza kuchanganuwa maswali au kuyabadili ule mfuatano, akizingatia majibu ya washiriki.**

1. Procedures

**(Utaratibu)** **Jinsi ya kufanya**

*Participant Characteristics*

**Sifa za mshiriki**

Before the interview begins, the researcher should record information on the participant’s age, gender, marital status (married monogamous, married polygynous, cohabitating, or unmarried), what they do for a living, and the number of children they have on the IDI reporting form. The participant’s name should not be recorded. Ensure the participant has provided consent prior to start of the IDI, including to audio record the session.

**Kabla ya kuanza mahojiano, mtafiti anatakiwa kuandika taarifa za mshiriki kuhusu umri wake, jinsia, hali ya ndoa ( ndoa ya mtu mmoja, ndoa ya wake wengi, kuishi pamoja na mwanaume bila ndoa, au hajaolewa), ni nini wanachofanya kwenye maisha, na idadi ya watoto walionao. Jina la mshiriki halitaandikwa. Hakikisha mshiriki ametoa ridhaa yake kabla ya kuanza mahojiano ya kina, pamoja na kunasa mazungumzo kwenye chombo husika.**

*Introduction*

**Utangulizi**

Greet the interviewee and give them an overview of the project, its aims, and their role in the outcomes of the study. This should be done by reading the participant **consent form**. After you read the form, please give a copy to the participant.

**Msalimie mhojiwa na muelezee kuhusu mtazamo wa mradi, makusudio yake na majukumu yake katika matokeo ya utafiti. Hii ingefanyika kwa kusoma fomu ya ridhaa ya mshiriki. Baada ya kumaliza kusoma fomu ya ridhaa, mpe mshiriki nakala yake.**

Please remind the participant that if they do not feel comfortable responding to certain questions, they do not have to respond. Their name will not be recorded and it will not be used in the notes from this recording or any other reporting. Tell the participant that they have been selected because we are interested in knowing their views; there are not wrong or right answers.

**Tafadhari mkumbushe mshiriki kuwa endapo hatajisikia vizuri kujibu baadhi ya maswali, basi asiyajibu. Jina lake halitaandikwa na halitatumika kwenye taarifa hizi au kwenye taarifa zingine. Mwambie mshiriki kuwa alichaguliwa kushiriki utafiti huu kwa sababu tunapenda kufahamu maoni yake; hakuna majibu sahihi au majibu mabaya.**

Ask what language the participant is most comfortable using (Sukuma or Swahili) and proceed accordingly.

**Mwulize mshiriki ni lugha gani angependa kuitumia (kisukuma au Kiswahili) na baadaye endelea.**

Tell the participant that you are happy to answer any questions they have about the research project now or at the end of the discussion. Ask “What are your questions now?” Answer any questions that arise.

Mwambie mshiriki kuwa **tuna furaha ya kujibu maswali yoyote atakayokuwa nayo kwa sasa na mwishoni mwa mazungumzo yanayohusu utafiti wa mradi. Mwulize “Je, una maswali ya kuuliza kwa sasa?” Jibu maswali yote yatakayojitokeza.**

Ask if it is ok to record this discussion. If it is ok, start recording and say “We are now ready to begin. I am now recording our conversation.”

**Mwulize kama itakuwa vizuri kunasa kwenye chombo mazungumzo haya. Kama itakuwa vizuri, anza kunasa mazungumzo na mwambie “kwa sasa tuko tayari kuanza. Nimeanza kunasa mazungumzo yetu”**

IF participant was part of FGD, it may be useful to start by referring back to the discussion:

*Paskazia, Dotto, and Eliza were all adolescents who were navigating moments of change in their lives. In our discussion the topic of marriage for Paskazia, Dotto, and Eliza came up and now we could like to discuss that more.*

***Paskazia, Dotto, and Eliza walikuwa wote ni vijana ambao wameelekeza mabadiliko ya muda mfupi kwenye maisha yao.***

1. Topic Guide

| **Topics** | **Main Questions** | **Follow-up Questions** | **Probes** |
| --- | --- | --- | --- |
| **Knowledge** | Can you tell me your thoughts on children below the age of 18 getting married? | In your view, is this something that happens here? What do you think about that? What have you heard from others in your community? | Do you know anything else about ‘child marriage’? |
| **Source of information** | Where did you hear about ‘child marriage’? | Who talks about ‘child marriage’ in this community? What do they say about it? | Can you give some examples? |
| **Experience** | Do you know anyone who married before age 18 years?  Did you marry before age 18 years? | How did the marriage come about? What did you/they feel about the marriage? What was the role of their/your parents? | Who influenced your/their decision? Why did you/they decide to marry when they did? How did you/they negotiate the disagreement with your/their parents (if disagreement was noted)? |
| **Opinion** | What do you think about ‘child marriage’? What are the advantages and disadvantages? | How do you think people in your community would react when someone marries before age 18 years? Do you think people want to marry early? Why or why not? | What are some ways an early marriage can harm or help a person? What about someone’s family? |

1. Mwongozo wa Mada

| **Mada** | **Maswali** | **Maswali ya ufuatiliaji** | **Hoji/chunguza zaidi** |
| --- | --- | --- | --- |
| **Elimu** | Kwa mawazo yako, unaweza kuniambia nini kuhusu watoto wanaoolewa wakiwa na umri chini ya miaka 18? | Kwa maoni yako, je, hiki ni kitu kinatokea na hapa. Je unafikiria nini kuhusu hilo? Je, umesikia nini kutoka kwa watu wengine katika jamii yako? | Je, unafahamu kitu kingine zaidi kuhusu ‘ndoa za utotoni’? |
| **Chanzo cha habari** | Je, wapi ulisikia kuhusu ‘ndoa za utotoni’? | Je, ni nani alizungumza kuhusu ‘ndoa za utotoni’ katika jamii hii? Je, walisemaje kuhusu hilo? | ‘Je, unaweza kunipa mifano? |
| **Uzoefu** | Je, unamfahamu mtu yeyote ambaye aliolewa kabla ya kufikisha miaka 18?  Je, wewe uliolewa kabla ya kufikisha umri wa miaka 18? | Je, ndoa ilikuwaje hatima yake? Je, ulijisikiaje/walijisikiaje kuhusu ndoa? Je, ni nini wajibu wa wazazi wako/wao? | Je, ni nani aliyekushawishi kuhusu maamuzi yako/yao? Je, kwa nini uliamua kuolewa/waliamua kuolewa wakati walipotaka? Je, ni kwa jinsi gani ulifanya/walifanya majadiliano katika kuondoa ugomvi au hali ya kutopatana na wazazi wako/wao (Kama ugomvi au hali ya kutopatana ilijulikana)? |
| **Maoni** | Je, unafikiri nini kuhusu ‘ndoa za utotoni’? Je, ni nini faida au hasara yake? | Je ni kwa kiasi gani unafikiri watu katika jamii yako wanavyolichukulia suala la mtu kuolewa kabla ya kufikisha umri wa miaka 18? Je unafikiri watu wanapenda kuolewa mapema? Kwa nini au kwa nini hapana? | Je, ni njia zipi ambazo ndoa za mapema zinaweza kuleta madhara au kumsaidia mtu?? Je, kwa familia nyingine hali iko vipi? |

**In-depth Interview**

**Reporting Form**

**Terehe: ________________________ Saa: _____________________**

**Jina ya umtafiti/watafiti: __________________________________________**

**Mshiriki:**

- Leader – Male
  - ***Role: _________***
- Respected woman (kama Balozi)
  - ***Role*:_____________________**
- Wazazi wa wanawake na wasichana
- Mwanamke wenye miaka 15- 24 – unmarried
- Mwanamke wenye miaka 15-24 – married
- Manaume wenye miaka 20-30

**Umri: _________**

**Idadi ya watoto: _______________**

**Wasichana? Ndiyo Hapana**

**Married? Ndiyo Hapana**

**Kama ndiyo:**

**Polygynous (or husband has other wives)**

**Monogamous (or husband has no other wives)**

**Kazi yako? ________________________________**

**Consent has been given:**

**Sahini:____________________**

**Summary of key points (conceptual points and any problems):**

|  |
| --- |
